# Hydrated Electrons in Phase-Matching Generation of Second-Order Stokes X-Waves in Water

**DOI:** 10.3390/molecules30091969

**Published:** 2025-04-29

**Authors:** Xinxin Chen, Qing Zhou, Zhongyang Wang

**Affiliations:** 1 Shanghai Advanced Research Institute, Chinese Academy of Sciences, Shanghai 201210, China; chenxinxin@sari.ac.cn (X.C.); zhouq@sari.ac.cn (Q.Z.); 2 University of Chinese Academy of Sciences, Beijing 100049, China

**Keywords:** second-order Stokes, X-waves, hydrated electrons, four-wave mixing, phase mismatch

## Abstract

Two components of X-waves, near-axis and off-axis, were observed in the generation of second-order Stokes around 550 nm, excited by intense 400 nm, 100 fs pump pulses in a 50 cm water cuvette. The emission angles of these two X-waves exhibited different evolutions; when the pump energy increased, the emission angle of the near-axis X-wave increased, while that of the off-axis X-wave decreased. These abnormal features of second-order X-waves came from the four-wave mixing process, accompanied by induced intense hydrated electrons via cascade ionization. The induced wave vector from high-density hydrated electrons led to angle-dependent phase-matching for the generation of the off-axis X-wave. However, for the generation of the near-axis X-wave, the induced wave vector from hydrated electrons initially compensated for the phase mismatch at a low pump energy, but as the energy increased, the phase mismatch also increased. Moreover, anomalous Raman shifts at second-order Stokes wavelengths (3262 cm^−1^ and 3350 cm^−1^) exhibited a similar evolutionary process to the anomalous Raman peaks at the Stokes wavelengths. The shifts arose from excess electrons being injected into the hydrogen bond network of water clusters.

## 1. Introduction

Stein [1] proposed in 1952 that hydrated or solvated electrons could exist in solids and liquids, while in 1962, Hart and Boag [2] first observed the characteristic transient absorption spectrum of hydrated electrons. Since then, hydrated electrons have been widely studied because of their significance in physical chemistry [3,4], photochemistry [5], and radiation chemistry [6,7]. With the advancement of ultrashort lasers, the solvation process [8,9,10,11], reaction mechanisms [12,13,14], relaxation dynamics [15,16,17,18,19], and transient spectral features [20,21,22] of hydrated electrons excited by ultrashort pulses have been widely studied. Although the formation process of hydrated electrons has been extensively studied [23,24], how the excess electrons change the hydrogen bonding networks of water clusters remains unclear. Yui et al. [25,26,27] observed anomalous Raman shifts at 3270 cm^−1^ and 3360–3380 cm^−1^ in backward-stimulated Raman scattering (SRS) by using intense 40 ps, 532 nm pulses focused into water. They assigned the appearance of anomalous Raman peaks due to the formation of the hydrated electrons. After laser pulse excitation, dense excess electrons are generated by the pulse front of the picosecond pulse, and the bonding network of water clusters are then changed as the OH moiety from each of several H_2_O molecules are preferably directed to the excess electron by hydrogen bonds [28]. Such highly polarized hydrated electrons led to the observation of anomalous Raman shifts in backward SRS. The presence of two distinct abnormal peaks suggests the existence of at least two different interactions between the OH groups and the excess electrons. Similarly, Tang and Wang [29] observed anomalous Raman peaks at 3260 cm^−1^ and 3355 cm^−1^ in forward SRS using a strong 2.5 ps, 400 nm pulse focused into water. In particular, when increasing the excitation, the saturation of hydrated electrons was observed. Correspondingly, the intensity of the anomalous Raman peak at 3260 cm^−1^ increased with an increase in the excitation and then became dominant in saturation excitation. The authors attributed this to the fact that the two anomalous Raman peaks arose from strongly bound hydrated electrons for 3260 cm^−1^ and weakly bound excess electrons (not fully hydrated) for 3355 cm^−1^.

High-power laser pulses propagating through a condensed media undergo space–time self-focusing, leading to the formation of filaments [30,31]. It has been established that one of the key signatures of filaments in normally dispersive condensed media is the generation of X-waves [32]. Filamentation has been shown to strongly enhance nonlinear interactions, such as four-wave mixing (FWM) [33] and SRS [34]. Faccio et al. [35] experimentally observed FWM-generated X-waves at the second-order Stokes wavelengths in water, which appeared only when the Stokes pulse was amplified. Correlated with Raman Stokes X-wave formation, the second-order Stokes X-waves arose from a phase-matched FWM process involving a pump, Stokes, and second-order Stokes X-waves. Furthermore, Faccio et al. [36] observed coherent anti-Stokes X-waves induced by FWM, which were found to exist at two distinct radiation angles. They explained this phenomenon using two different types of phase-matching conditions: non-transversal phase-matching and perfect phase-matching.

In this article, we report the observation of two components of X-waves (near-axis and off-axis) in the generation of second-order Stokes waves by exciting 50 cm water with intense 100 fs, 400 nm pump pulses. With increases in the excitation intensity, the emission angle increased for the X-wave of the near-axis component, while the emission angle decreased for the X-wave of the off-axis component, indicating different phase-matching conditions for the two X-waves. We found that the excitation-intensity-dependent cascade generation of hydrated electrons was the origin of such abnormal phenomena. The induced wave vector from such excitation-dependent hydrated electrons led to angle-dependent phase-matching for the generation of the off-axis X-wave, while first causing the compensation of the phase mismatch at a low pump energy and then increasing the phase mismatch at a high pump energy for the generation of the near-axis X-wave. Consequently, when the hydrated electron density reached saturation, the off-axis X-wave became approximately 2.3 times stronger than the near-axis X-wave. The spectrum of both components exhibited anomalous Raman shifts, which again shows the key role of hydrated electrons. At a low excitation intensity, a weak second-order Stokes emission around 550 nm was observed, corresponding to a Raman shift of 3400 cm^−1^, which was associated with the ordinary OH stretching vibrations of water molecules [25]. As the excitation intensity increased, a forward Raman emission emerged around 544 nm, corresponding to anomalous Raman shifts at 3262 cm^−1^ and 3350 cm^−1^. The appearance of these anomalous Raman shifts corresponded to the production of hydrated electrons, suggesting that they result from changes in the hydrogen bond structure.

## 2. Results

### 2.1. The Nonlinear Second-Order Stokes X-Waves in Water

**Figure 1:**
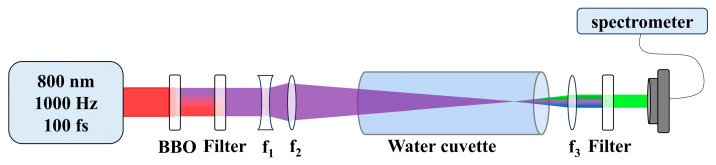
Schematic of the experiment setup. BBO: second-harmonic crystal, f: focusing lens.

Specific experimental details were shown in Figure 1. When the input energy exceeded 5 μJ, filaments began to appear. In our previous experiments, by adjusting the focus to 40 cm and setting the pump energy to 70 μJ, we maximized both the length of filaments and the Stokes conversion efficiency. We attributed it to group velocity matched stimulated Raman conversion between the pump and Stoke X-waves [37]. However, the generation of accompanying second-order Stokes has not yet been studied. In this article, we will explore the abnormal phenomena of second-order Stokes generation accompanied by filaments.

**Figure 2:**
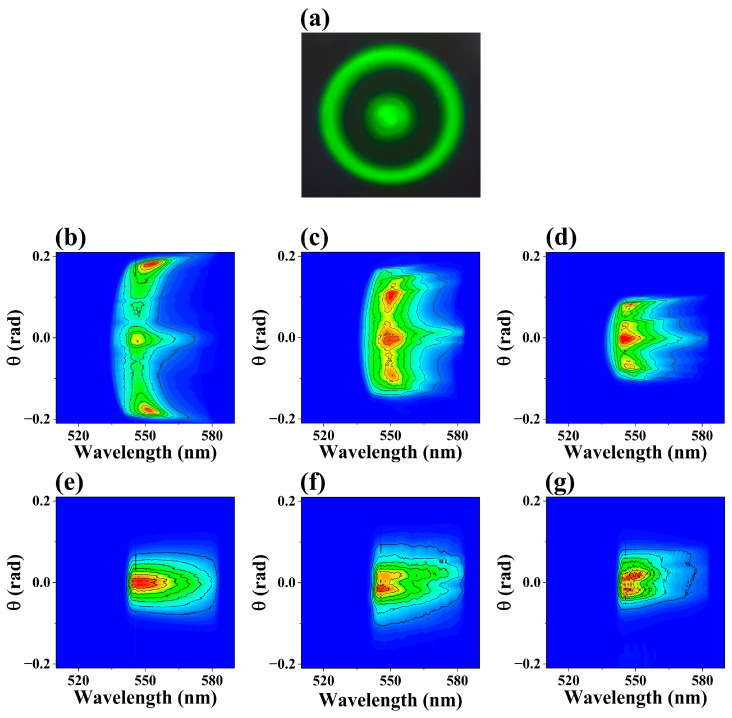
(**a**) The conical emission around 550 nm when the input energy exceeds 15 μJ, consisting of a central spot and an outer ring. This emission was observed on a piece of paper in front of spectrometer. The measured (θ, λ) spectrum of the two components of X-waves (near-axis and off-axis) in the generation of second-order Stokes, centered around 550 nm, were shown for various input energies: (**b**,**e**) E_in_ = 15 μJ, (**c**,**f**) E_in_ = 30 μJ, (**d**,**g**) E_in_ = 50 μJ. Here, (**a**–**c**) show the (θ, λ) spectrum of both off-axis and near-axis X-waves, (**d**–**g**) show the (θ, λ) spectrum of the near-axis X-wave only.

**Figure 3:**
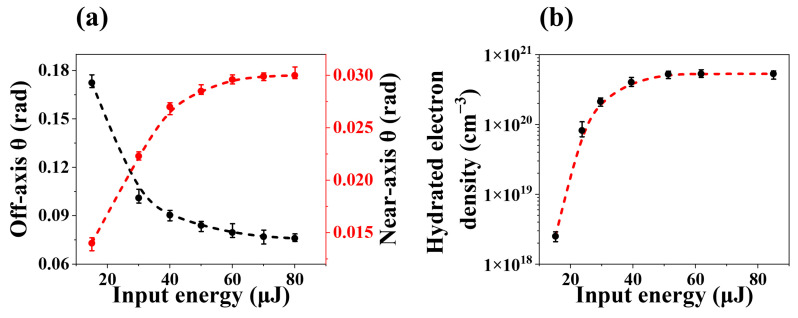
(**a**) The emission angles of the second-order Stokes X-waves, for both near-axis (red dots) and off-axis (black dots), change with increasing pump energy. (**b**) The measured hydrated electron density with the increasing pump energy in water.

To study the second-order Stokes, we filtered out the pump and Stokes wavelengths after the water cuvette. A conical emission was observed in front of spectrometer when the input energy exceeded 15 μJ, consisting of a central spot and an outer ring around 550 nm, as shown in Figure 2a. Their (θ, λ) spectrum at various pump energies were shown in Figure 2b–d, where the near-axis and off-axis X-waves corresponded to the central spot and the outer ring, respectively. To clearly describe the characteristics of the near-axis X-wave, the (θ, λ) spectrum at various pump energies were recorded by blocking the outer ring, as shown in Figure 2e,f. With the increasing pump energy, the emission angles of these two X-waves displayed different evolutions. The emission angle of the near-axis X-wave increased, while that of the off-axis X-wave decreased. It was again shown in Figure 3a that the emission angles of the X-waves (near-axis and off-axis) exhibited different trends as the pump energy increased. For pump energies of 15 μJ, 30 μJ, 40 μJ, 50 μJ, 60 μJ, 70 μJ and 80 μJ, the emission angle of the near-axis X-wave gradually increased, reaching 0.0140 rad, 0.0223 rad, 0.0270 rad, 0.0285 rad, 0.0296 rad, 0.0299 rad, 0.0300 rad, respectively. In contrast, the emission angle of the off-axis X-wave gradually decreased, corresponding to 0.172 rad, 0.101 rad, 0.0904 rad, 0.0840 rad, 0.0796 rad, 0.0770 rad and 0.0760 rad, respectively. Both the near-axis and off-axis X-waves emission angles approached stability at a pump energy of 60 µJ. Thus, we can conclude that the on-axis and off-axis emissions represented different X-waves in the generation of second-order Stokes.

Our previous measurements revealed that the pulse width had been stretched to 2–2.8 ps, depending on the input energy at 40 cm focus after 100 fs pulses weakly focused into the 50 cm water cuvette. With picosecond pulse widths and input energy above the Kerr threshold, the cascade generation of hydrated electrons was dominant, leading to induced densities that were orders of magnitude higher than those excited by hundreds femtosecond pulses [37,38,39]. In order to show how our observed phenomena correlated with the density of hydrated electrons, we measured the densities of hydrated electrons as a function of the pump energy. We used stretched 2.5 ps, 400 nm pump pulses to excite a 1 cm water cuvette, and probed at the absorption peak of hydrated electrons using 100 fs, 720 nm pulses. Details of the experimental setup can be found elsewhere [29].The hydrated electron density was derived from the induced transient absorption signals using the Lambert-Beer law ($A=\sigma_{a}\rho z_{R}/\ln10$), where $\sigma_{a}=1.4\times 10^{-18}{\mathrm{cm}}^{2}$ [40] is the absorption section of hydrated electron, $z_{R}$ is the Raleigh range. As shown in Figure 3b, we observed a nonlinearly increase in hydrated electron density as the pump energy increased from 10 μJ to 40 μJ, approaching saturation after 40 μJ and reaching saturation around 60 μJ, which was highly correlated with the rapid change in the emission angle observed from 10 μJ to 40 μJ, stabilizing after 40 μJ remaining stable at 60 μJ. Both the near-axis and off-axis X-waves exhibited this trend, indicating that both of them were highly influenced by dense hydrated electrons.

### 2.2. The Conversion Efficiency of Second-Order Stokes in Water

**Figure 4:**
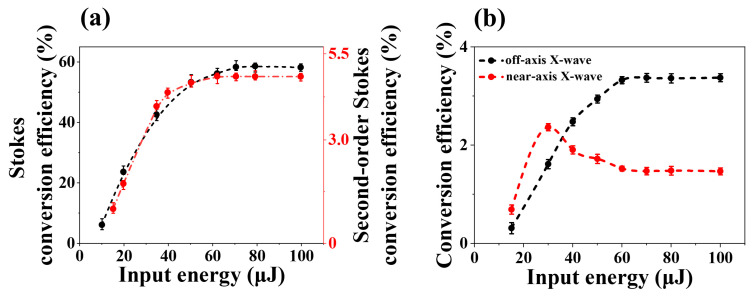
(**a**) The measured Stokes conversion efficiency (black dots) and second-order Stokes conversion efficiency (red dots) with the increasing input pump energy. (**b**) The conversion efficiency of off-axis X-wave (black dots) and near-axis (red dots) X-wave at the second-order Stokes with increasing input pump energy.

We measured the conversion efficiencies of Stokes and second-order Stokes as the input pump pulse energy increased, as shown in Figure 4a. These conversion efficiencies were measured by filtering out either only the 400 nm pump pulse or both the 400 nm pump pulse and the 460 nm Stokes pulse, respectively. When the input pump pulse energy exceeded 5 μJ, the Stokes conversion efficiency (black dots) increased nonlinearly and saturated at around 70 µJ with an efficiency of approximately 60%. Similarly, when the input pump pulse energy exceeded 15 µJ, the second-order Stokes conversion efficiency (red dots) also exhibited nonlinear growth, reaching saturation at around 60 µJ with a value of about 5%. The second-order Stokes conversion efficiency saturated before the Stokes, indicating that the generation of second-order Stokes also relied on the pump energy via a FWM process.

As we observed two distinct evolutionary trends of the second-order Stokes X-waves, near-axis and off-axis, in Figure 2, we measured their conversion efficiencies separately, as shown in Figure 4b. At the beginning of 15 μJ pump energy, the intensity of the near-axis X-wave was approximately twice that of the off-axis X-wave. As the input energy increased, the intensity of the near-axis X-wave first approached a peak around 30 μJ and then decreased. However, the intensity of the off-axis X-wave gradually rose and eventually became dominant. The conversion efficiencies of both second-order X-wave components approach stable at 60 μJ, with the off-axis X-wave becoming approximately 2.3 times stronger than the near-axis X-wave.

### 2.3. The Forward Second-Order Stokes Spectrum in Water

**Figure 5:**
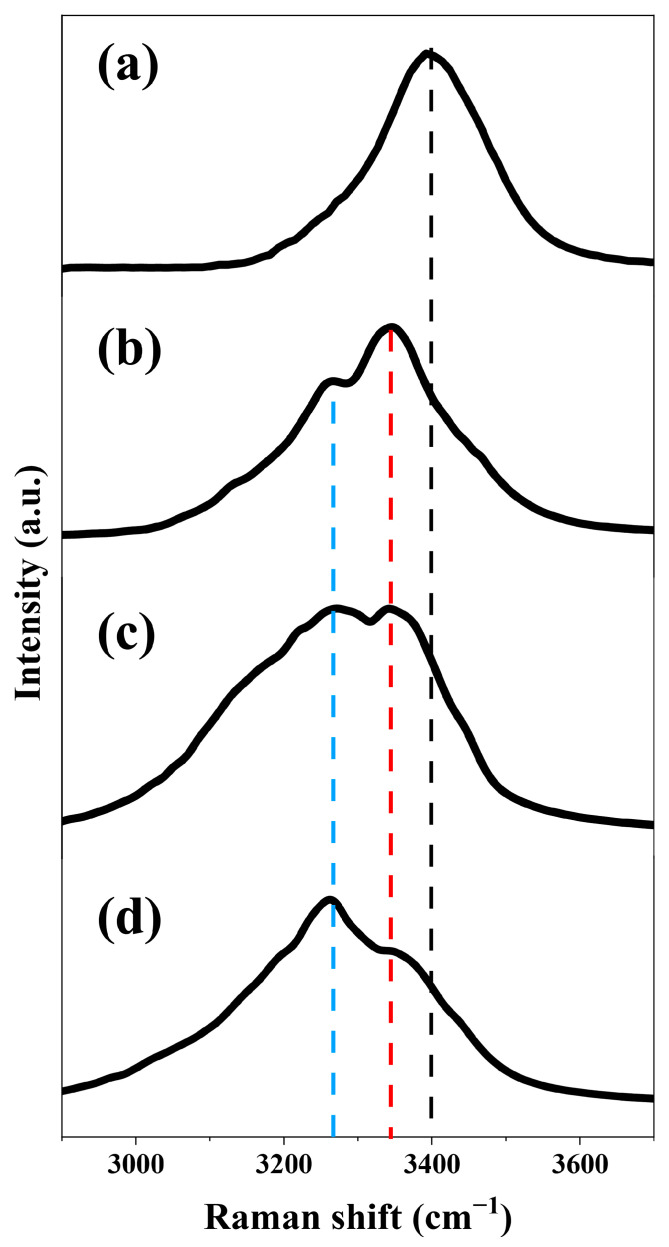
Measurement of forward second-order Stokes emission with different input pump energies in distilled water. (**a**) The Raman shifts of the second-order Stokes at 3400 cm^−1^ (black dashed line) with an input energy of 10 μJ. As the input pump energy increased to (**b**) 15 μJ, (**c**) 30 μJ, (**d**) 45 μJ, two distinct peaks in the forward second-order Stokes emission emerged at 3262 cm^−1^ (blue dashed line) and 3350 cm^−1^ (red dashed line), respectively. The black dashed line represents the Raman shift at 3400 cm^−1^, corresponding to the characteristic OH stretching vibration of water molecules in the liquid phase.

Based on the previous analysis, we reported two components of X-waves (near-axis and off-axis) in the generation of second-order Stokes, which resulted from FWM along with high-density hydrated electrons in water. We measured the Raman spectrum of these two components at various input energies and found that their Raman shifts also change with the pump energies, as shown in Figure 5.

When the input energy was set to 10 μJ, we observed second-order Stokes emission around 550 nm, corresponding to a Raman shift of 3400 cm^−1^, as shown in Figure 5a. This shift was associated with the ordinary OH stretching vibrations of water molecules [25]. The FWHM of the ordinary Raman shift in the vibrational band structure of water clusters was about 190 cm^−1^, which was nearly identical to the typical SRS linewidth in water [41]. When the input energy exceeded 15 μJ, a forward Raman emission was observed around 544 nm, corresponding to anomalous Raman shifts at 3262 cm^−1^ (blue dashed line) and 3350 cm^−1^ (red dashed line), as shown in Figure 5b–d. For input energies of 15 µJ, 30 µJ and 45 µJ, the FWHM of the anomalous Raman shifts were 256 cm^−1^, 312 cm^−1^ and 322 cm^−1^, respectively. With an input energy set to 15 μJ, a strong peak at 3350 cm^−1^ and a weak peak at 3262 cm^−1^ were observed. With an input energy of 45 μJ, the intensity of 3262 cm^−1^ exceeded that of the 3350 cm^−1^ peak. The intensity of the 3262 cm^−1^ peak became stronger relative to that of the 3350 cm^−1^ peak as the input energy increased.

The anomalous Raman shifts we observed at the second-order Stokes wavelengths exhibited a similar evolutionary process to the previously reported anomalous Raman peaks of Stokes wavelengths at 3260 cm^−1^ and 3355 cm^−1^, observed by Tang and Wang [29]. Therefore, similar to the abnormal Raman shift at Stokes, the anomalous Raman shift at the second-order Stokes also arose from excess electrons being injected into the hydrogen bond network of water clusters. This process altered the hydrogen bond network structure and the clustering dynamics of water. We believe that the excess electrons exhibited two forms, fully hydrated and not fully hydrated (or free) electrons, which contributed to the anomalous Raman peaks at 3262 cm^−1^ and 3350 cm^−1^, respectively. When the hydrated electrons saturated, the contribution of the 3262 cm^−1^ peak became dominant, as we observed in the experiment.

## 3. Discussion

As the experiment has already shown, our observed phenomena were highly correlated with the induced hydrated electrons. The abnormal features of second-order X-waves came from FWM process along with highly induced excess electrons. Such excess electrons changed the phase-matching conditions of the generation of both near-axis and off-axis X-waves; however, their influence was different.

**Figure 6:**
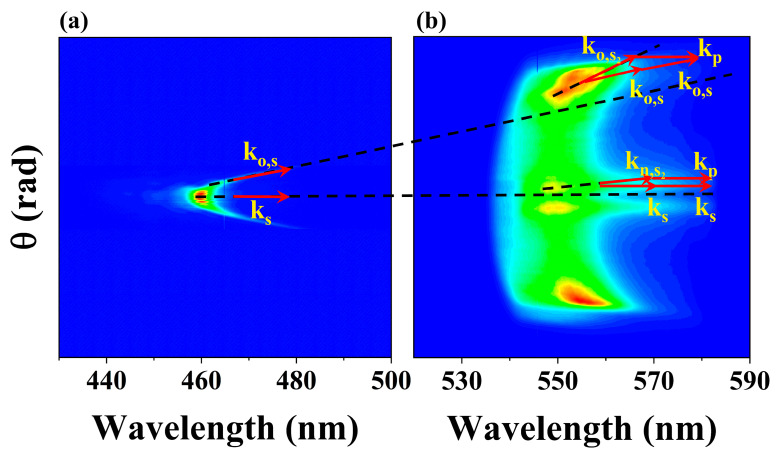
(**a**) The (θ, λ) spectrum of X-wave in the generation of Stokes with the input energy of 50 μJ, centered around 460 nm. The red arrows represented the Stokes wave vector along the axis *k_s_* and the off-axis tail of X-wave *k_o,s_*, respectively. (**b**) The (θ, λ) spectrum of the two components of X-waves (near-axis and off-axis) in the generation of second-order Stokes with the input energy of 50 μJ, centered around 550 nm. The two inserts showed the two different phase-matching conditions, respectively. For the off-axis X-wave, the FWM process involved the on-axis pump pulse *k_p_*, the off-axis tail of the Stokes X-wave *k_o,s_*, and the off-axis second-order Stokes X-wave *k_o,s__2_*. For the near-axis X-wave, the FWM process involved the on-axis pump pulse *k_p_*, the on-axis Stokes X-wave *k_s_*, and the near-axis second-order Stokes X-wave *k_n,s__2_*.

When the input pump energy was set at 15 μJ, the two components of second-order Stokes X-waves began to appear around 550 nm, while the intensity of the near-axis X-wave approximately twice that of the off-axis X-wave. In this case, the hydrated electron density ($\rho \approx 2\times 10^{18}{\mathrm{cm}}^{-3}$) was relatively low, approximately two orders of magnitude lower than the maximum density achieved at 60 µJ pump energy, as shown in Figure 3b. This low density of electrons changed the phase match condition little. As the pump energy increased, the emission angle of the near-axis X-wave increased, while that of the off-axis X-wave decreased. Additionally, the intensity of the near-axis X-wave initially approached a peak around 30 μJ before decreasing, whereas the intensity of the off-axis X-wave gradually rose and eventually dominated. Meanwhile, the density of hydrated electrons exhibited nonlinear growth and saturated at 60 μJ ($\rho \approx 5\times 10^{20}{\mathrm{cm}}^{-3}$). These results indicated that the high-density of hydrated electrons was beneficial to the off-axis phase-matching, while causing the increase of phase mismatch for the generation of the near-axis X-wave. To better explain the two distinct phase-matching conditions, a phase-matching schematic diagram (Figure 6) was shown with the input energy of 50 μJ. The (θ, λ) spectrum of X-wave in the generation of Stokes with the input energy, centered around 460 nm, was shown in Figure 6a, and the red arrows represent the Stokes wave vector along the axis *k_s_* and the off-axis tail of X-wave *k_o,s_*, respectively. The (θ, λ) spectrum of the two components of X-waves (near-axis and off-axis) in the generation of second-order Stokes with the input energy of 50 μJ, centered around 550 nm was shown in Figure 6b. The two inserts showed the two different phase-matching conditions, respectively.

For the off-axis X-wave component in the generation of second-order Stokes, we attributed the phase-matching in this case to a FWM process involving the on-axis pump pulse, the off-axis tail of the Stokes X-wave, and the off-axis second-order Stokes X-wave, as shown in the insert in Figure 6b. As the pump energy increased, the high electron density compensated for the group delay of Stokes, leading to a smaller radiation angle of the off-axis tail. Consequently, the off-axis X-wave angle of second order Stokes decreased as the pump energy increased. The total phase mismatch $△k_{o}$ for the generation of the off-axis second-order Stokes X-wave along the propagation z axis can be expressed as

(1) $$△k_{o}=△k_{o,water}+△k_{o,eq^{-}},$$

where $△k_{o,water}$ resulting from the phase mismatch from dispersion of water, and $△k_{o,eq^{-}}$ resulting from the phase mismatch by the induced hydrated electrons, here *o* represents the off-axis. According to the FWM process, $△k_{o,water}$ can be expressed as

(2) $$△k_{o,water}=2k_{o,s,water}-k_{p,water}-k_{o,s_{2},water},$$

where $k_{i}=n\omega_{i}/c,i=p,s,s_{2}$ is wave vector for pump, Stokes and second-order Stokes, respectively. The $△k_{o,eq^{-}}$ can be expressed as

(3) $$△k_{o,eq^{-}}=2k_{o,s,eq^{-}}-k_{p,eq^{-}}-k_{o,s_{2},eq^{-}},$$

the induced wave vector from electrons can be expressed as $k_{eq^{-}}=\omega_{i}△n_{eq^{-}}/c$, where $\Delta n_{eq^{-}}=-\omega_{eq^{-}}^{2}/2n_{0}\omega_{i}^{2}$ is the induced refractive index, $\omega_{eq^{-}}^{2}=\rho e^{2}/\varepsilon_{0}m$ is the induced plasma frequency, and ρ is the electron density [42]. With the pump energies of 15 µJ, 30 µJ, 40 µJ, 50 µJ, 60 µJ, 70 µJ, and 80 µJ, the phase mismatch from water were $△k_{o,water}\approx (1.78\times 10^{5},6.74\times 10^{4},5.59\times 10^{4},4.95\times 10^{4},4.54\times 10^{4},4.31\times 10^{4},4.22\times 10^{4})$ m^−1^, and the phase mismatch from electrons were $△k_{o,eq^{-}}\approx -(1.12\times 10^{2},1.27\times 10^{4},2.47\times 10^{4},3.26\times 10^{4},3.33\times 10^{4},3.34\times 10^{4},3.35\times 10^{4})$ m^−1^, and the total phase mismatch gives $△k_{o}\approx (1.78\times 10^{5},5.47\times 10^{4},3.12\times 10^{4},1.69\times 10^{4},1.21\times 10^{4},9.65\times 10^{3},8.71\times 10^{3})$ m^−1^, respectively. Therefore, the high-density electrons promoted phase-matching in the generation of the off-axis X-wave. This explains the observed increase in the relative intensity of the off-axis X-wave with increasing input energy in Figure 4b.

For the near-axis X-wave component, we attributed the phase-matching in this case to a FWM process involving the on-axis pump pulse, the on-axis Stokes, and the near-axis second-order Stokes X-wave, as shown in the insert in Figure 6b. The phase mismatch for the near-axis X-wave in the generation of second-order Stokes can be expressed as

(4) $$△k_{n}=△k_{n,water}+△k_{n,eq^{-}}$$

where *n* represents the near-axis. According to the FWM process, $△k_{n,water}$ can be expressed as

(5) $$△k_{n,water}=2k_{s,water}-k_{p,water}-k_{n,s_{2},water},$$

and $△k_{n,eq^{-}}$ can be expressed as

(6) $$△k_{n,eq^{-}}=2k_{s,eq^{-}}-k_{p,eq^{-}}-k_{n,s_{2},eq^{-}}.$$

With the pump energies of 15 µJ, 30 µJ, 40 µJ, 50 µJ, 60 µJ, 70 µJ, and 80 µJ, the phase mismatch from water were $△k_{n,water}\approx (1.04\times 10^{4},1.21\times 10^{4},1.34\times 10^{4},1.39\times 10^{4},1.42\times 10^{4},1.44\times 10^{4},1.44\times 10^{4})$ m^−1^, the phase mismatch from electrons were $△k_{n,eq^{-}}\approx -(1.67\times 10^{2},1.01\times 10^{4},2.65\times 10^{4},3.43\times 10^{4},3.45\times 10^{4},3.44\times 10^{4},3.44\times 10^{4})$ m^−1^, and the total phase mismatch gives $△k_{n}\approx (1.02\times 10^{4},1.98\times 10^{3},-1.31\times 10^{4},-2.04\times 10^{4},-2.03\times 10^{4},-2.01\times 10^{4},-2.00\times 10^{4})$ m^−1^, respectively. At low pump energies from 15 µJ to 30 µJ, the induced phase mismatch from electrons compensated for the phase mismatch from the dispersion of water, leading to an increase in the conversion efficiency of the near-axis X-wave. However, with further increases in the pump energy, the induced phase mismatch from electrons became larger than the phase mismatch from the dispersion of water, resulting in a decrease in conversion efficiency. In this case, the increased emission angle of the near-axis X-wave helped to partly compensate the phase mismatch.

As a result, we found two phase-matching configurations involved in the generation of second-order Stokes X-waves. The induced wave vector from high-density electrons led to angle-dependent phase-matching for the generation of the off-axis X-wave, while it first compensated for the phase mismatch at low pump energy and then increased the phase mismatch at high pump energy for the generation of the near-axis X-wave.

## 4. Experiments

A schematic of the experimental setup was shown in Figure 1. The output pulses from a Ti:Sapphire regeneration system (800 nm, 100 fs, 3.5 mJ, 1000 Hz) were frequency-doubled to 400 nm using a BBO crystal, producing pulses with energies ranging from 5 to 200 µJ per pulse. These pulses were used as the pump pulses. A pair of lenses (f_1_ = −75 mm and f_2_ = 125 mm) was used to weakly focus the laser pulses into a 50 cm water cuvette (placed 10 cm behind the lens f_2_), which was made of polyvinyl chloride and equipped with two fused silica windows. The focal diameter of the pulses in water was measured as 38.5 ± 1.6 μm. After passing through the water, the output forward signals emitted from the water cuvette were collected by a lens (f_3_ = 75 mm). A band-pass filter with a cut-on wavelength of 550 ± 40 nm (FBH 550-40) was used to filter out the 400 nm pump pulse and the 460 nm Stokes pulse. A spectrometer with a CCD (Horiba iHR320, Kyoto, Japan) was then used to image the far-field angle-wavelength (θ, λ) spectrum of the output signals. All experiments were performed at ambient temperature and pressure.

## 5. Conclusions

In this study, we observed two abnormal components of X-waves in the generation of second-order Stokes by using an intense 400 nm, 100 fs pump pulse weakly focused into to a 50 cm water cuvette. As the input energy increased, the emission angles and energy conversion efficiencies of the near-axis and off-axis X-waves exhibited distinct trends: the emission angle of the near-axis increased, while that of the off-axis decreased. Based on these observations, we found two different phase-matching configurations for the FWM process dominated by hydrated electrons. The induced wave vector from high-density electrons led to angle-dependent phase-matching for the generation of the off-axis X-wave, while it first compensated for phase mismatch at low pump energy and then increased the phase mismatch at high pump energy for the generation of the near-axis X-wave. As a result, when the hydrated electron density reached saturation, the off-axis X-wave component became approximately 2.3 times stronger than the near-axis X-wave component. Additionally, we observed the same anomalous Raman shifts at the second-order Stokes emission for both components, similar to those in the Stokes emission, at 3262 cm^−1^ and 3350 cm^−1^. These shifts can be attributed to the production of hydrated electrons, suggesting that they result from changes in the hydrogen bond structure.

## Data Availability

Data are available on request due to restrictions, e.g., privacy or ethics.
